# Tuberculosis among people living with HIV/AIDS in the German ClinSurv HIV Cohort: long-term incidence and risk factors

**DOI:** 10.1186/1471-2334-14-148

**Published:** 2014-03-19

**Authors:** Basel Karo, Walter Haas, Christian Kollan, Barbara Gunsenheimer-Bartmeyer, Osamah Hamouda, Lena Fiebig

**Affiliations:** 1 Department for Infectious Disease Epidemiology, Robert Koch Institute, Seestr. 10, 13353 Berlin, Germany; 2 Berlin School of Public Health, Charité – Universitätsmedizin Berlin, Berlin, Germany

**Keywords:** Epidemiology, Incidence, HIV/AIDS, Tuberculosis, Coinfection, Antiretroviral therapy, Isoniazid preventive therapy, Industrialized country, Germany, Immigration

## Abstract

**Background:**

Tuberculosis (TB) still presents a leading cause of morbidity and mortality among people living with HIV/AIDS (PLWHA), including those on antiretroviral therapy. In this study, we aimed to determine the long-term incidence density rate (IDR) of TB and risk factors among PLWHA in relation to combination antiretroviral therapy (cART)-status.

**Methods:**

Data of PLWHA enrolled from 2001 through 2011 in the German ClinSurv HIV Cohort were investigated using survival analysis and Cox regression.

**Results:**

TB was diagnosed in 233/11,693 PLWHA either at enrollment (N = 62) or during follow-up (N = 171). The TB IDR during follow-up was 0.37 cases per 100 person-years (PY) overall [95% CI, 0.32-0.43], and was higher among patients who never started cART and among patients originating from Sub-Saharan Africa (1.23 and 1.20 per 100PY, respectively). In two multivariable analyses, both patients (I) who never started cART and (II) those on cART shared the same risk factors for TB, namely: originating from Sub-Saharan Africa compared to Germany (I, hazard ratio (HR); [95% CI]) 4.05; [1.87-8.78] and II, HR 5.15 [2.76-9.60], CD4+ cell count <200 cells/μl (I, HR 8.22 [4.36-15.51] and II, HR 1.90 [1.14-3.15]) and viral load >5 log_10_ copies/ml (I, HR 2.51 [1.33-4.75] and II, HR 1.77 [1.11-2.82]). Gender, age or HIV-transmission risk group were not independently associated with TB.

**Conclusion:**

In the German ClinSurv HIV cohort, patients originating from Sub-Saharan Africa, with low CD4+ cell count or high viral load at enrollment were at increased risk of TB even after cART initiation. As patients might be latently infected with *Mycobacterium tuberculosis* complex, early screening for latent TB infection and implementing isoniazid preventive therapy in line with available recommendations is crucial.

## Background

The co-infection of tuberculosis (TB) and Human Immunodeficiency Virus (HIV) poses a major challenge to public health for both developing and industrialized countries [1]. According to WHO (2012) [2], an estimated 1.1 million new TB cases were reported among people living with HIV/AIDS (PLWHA). TB still presents a leading cause of morbidity and mortality among PLWHA, including those on antiretroviral therapy (ART) [3]. The greatest burden of TB/HIV co-morbidity was found in Sub-Saharan Africa, where approximately 79% of global TB/HIV cases occur [2]. Analogously, recent studies from Europe showed that Sub-Saharan African immigrants were at the highest risk of TB/HIV co-infection among the general population [4-6].

Germany is a low incidence country for TB and a low HIV-prevalence country. It has a population of 80.5 million of whom about 8% are foreign nationals and 20% are estimated to have a migration background, predominantly from Turkey, Poland and Newly Independent States of the Former Soviet Union [7]. The incidence of TB has continuously fallen in the general population, but the rate of decline has slowed in the last years (9.3, 7.3, 5.5 and 5.3 cases per 100,000 population in 2002, 2005, 2008 and 2011, respectively) [8]. In the same period, the number of newly diagnosed HIV cases has substantially increased (1,719 cases in 2002 vs. 2,889 cases in 2011) [9]. Individuals with foreign origin from high burden countries are considered as risk groups for both HIV and TB in Germany. Sub-Saharan Africa represents the main foreign region of origin among PLWHA. While, Turkey and the Newly Independent States of the former Soviet Union are the main foreign countries of birth among TB patients [8,9]. HIV prevalence in TB patients was estimated to be 4.5% in 2009 [10]. But due to the separate reporting pathways of TB and HIV in the national surveillance system [11], little is known on the TB/HIV burden and risk factors of TB among PLWHA in Germany. However, additional HIV surveillance instruments such as the German ClinSurv HIV Cohort include information on TB as an AIDS-defining disease.

The introduction of combination antiretroviral therapy (cART) in the mid-1990s has markedly reduced HIV-related morbidity and mortality [12,13]. The preventive impact of ART on HIV-associated TB can be attributed to suppression of viral replication, which permits both quantitative and functional reconstitution of the host’s immune system [12]. Although cART is associated with substantial declines in TB risk by 70%-90% among PLWHA [12], limitations of cART in reducing TB risk have been observed among some sub-populations such as elderly patients and patients with low CD4+ cell count in high-income countries [14]. A study conducted in London to investigate the effect of the initial cART on AIDS-related diseases over a 9-year period found no significant change in TB incidence before and after the introduction of cART [15]. Antiretroviral treatment might be also complicated by the immune reconstitution inflammatory syndrome (IRIS) in patients who start anti-HIV medications during TB treatment manifested by clinical deterioration in spite of increased CD4+ cell count and decreased plasma viral load [16].

Overall, little information is available on the risk profiles for TB among PLWHA living in Germany whether cART has been initiated or not.

With this study, we aimed to describe the characteristics of TB/HIV patients within the German ClinSurv HIV Cohort; to estimate the TB incidence density rate; and to identify factors associated with TB in PLWHA on cART and those who never started cART.

## Methods

### Data source and study population

The study was based on the German ClinSurv HIV Cohort, which is an ongoing open multicentre cohort for the clinical surveillance of HIV disease. The ClinSurv HIV cohort was established in 1999 as a collaborative cohort between specialized treatment centres for HIV/AIDS and the Robert Koch Institute (the National Public Health Institute of Germany). Irrespective of the disease stage, all HIV-infected patients are eligible to attend one of the treatment centres. After three consecutive days of clinical observation, patients will automatically be enrolled in the ClinSurv HIV Cohort. The ClinSurv dataset includes basic demographic data recorded anonymously at the first contact. Additionally, clinical, laboratory and medication history data are reported and updated at each follow-up contact. More details on the German ClinSurv HIV Cohort Study are provided by Baetzing-Feigenbaum et al. [17].

For the current analysis, data of patients enrolled in the cohort from January 2001 (introduction of the co-formulation boosted protease inhibitors Lopinavir/Ritonavir in Germany [18]) through December 2011 were extracted from the central ClinSurv HIV database. Basic demographic characteristics including age, gender and geographic origin are recorded as time-fixed covariates at the time of enrollment. Clinical assessments (diabetes mellitus, hepatitis B and C) are coded as binary covariates. Laboratory measurements (CD4+ cell counts and viral load) and medication information (TB regimens and type of ART) are coded as time-varying covariates updated in 3-month-periods when data is available.

Patients who received combination therapy involving two nucleoside reverse-transcriptase inhibitors (NRTI) plus one non-nucleoside reverse-transcriptase inhibitor (NNRTI) or protease inhibitor were defined as patients on cART. The remainder were classified as patients who never started cART during follow-up, who may however receive a different form of ART.

### TB/HIV case definition

TB cases were reported including date of diagnosis; patients diagnosed with TB prior to enrollment were excluded from the analysis. TB/HIV cases were defined as any form of TB in PLWHA confirmed by positive culture of *Mycobacterium tuberculosis* complex. TB in PLWHA is an AIDS defining disease; therefore all TB/HIV patients were in the C category according to the CDC classification system for the clinical categories of HIV infection [19].

### Statistical analysis

Continuous variables were described using medians with interquartile ranges (IQR) and compared by the Mann–Whitney *U*-test. Categorical variables were described using numbers and percentages and compared by χ^2^ test.

For the survival analysis, the observation period was calculated as the time from enrollment to either diagnosis of TB, last follow-up (follow-up contact was defined by at least one visit to center every 6 months) or the end of observation period (December 2011).

The TB incidence density rate (IDR) was defined as the number of TB cases occurring per 100 patient-years (PY) of observation. The TB IDR was further stratified by gender, age group, region of origin, HIV-transmission risk group, baseline CD4+ cell count, baseline viral load, diabetes mellitus, hepatitis infections and cART-status. *P*-value was obtained by χ^2^ test for difference in TB IDRs among different groups. To compare TB IDRs after controlling for region of origin and age, the Mantel-Haenszel test was applied. Trend analyses of TB incidence were conducted using the nonparametric test for trend across ordered groups.

To compare survival probabilities between patients on cART and those who never started cART, the observation period for patients on cART was modified to start at the date when the patient first received cART and before TB diagnosis. Baseline CD4+ cell count and viral load for patients on cART were set on the date of cART initiation instead of enrollment. The endpoints of the observation remained as defined in the survival analysis. We assumed that once started on cART, patients remained on it.

The Kaplan-Meier survivor function was used to estimate TB-free survival probabilities. TB-free survival was further stratified by demographic factors, CD4+ cell count and cART-status and was compared using log-rank tests. Two multivariable Cox proportional hazards regression models, one for patients on cART and another for those never starting cART were constructed to identify factors associated with TB in relation to cART-status. Independent variables (gender, age group, region of origin, HIV-transmission risk group, baseline CD4+ cell count and viral load for patients never started cART and CD4+ cell count and viral load fixed at the time of cART initiation for the patients on cART) with *P* < 0.02 in the log-rank test were included in the Cox regression models. The proportionality assumption of the final model was checked using the likelihood-ratio test and the Schoenfeld and Scaled Schoenfeld residuals. These applied tests indicated no violation of the model’s proportionality. Furthermore, the goodness of fit of the final model was evaluated by the Cox-Snell residuals.

All tests were two sided with 95% confidence interval (CI); the level of significance was *P* < 0.05. All analyses were performed using STATA (version12, StataCorp, LP, TX, USA) software.

### Ethical statement

The ClinSurv HIV study protocol was approved by the German Federal Commissioner for Data Protection and Freedom of Information who stated that written informed consent from the patients was not required due to the anonymous nature of the data.

## Results

### Characteristics of patients

**Figure 1:**
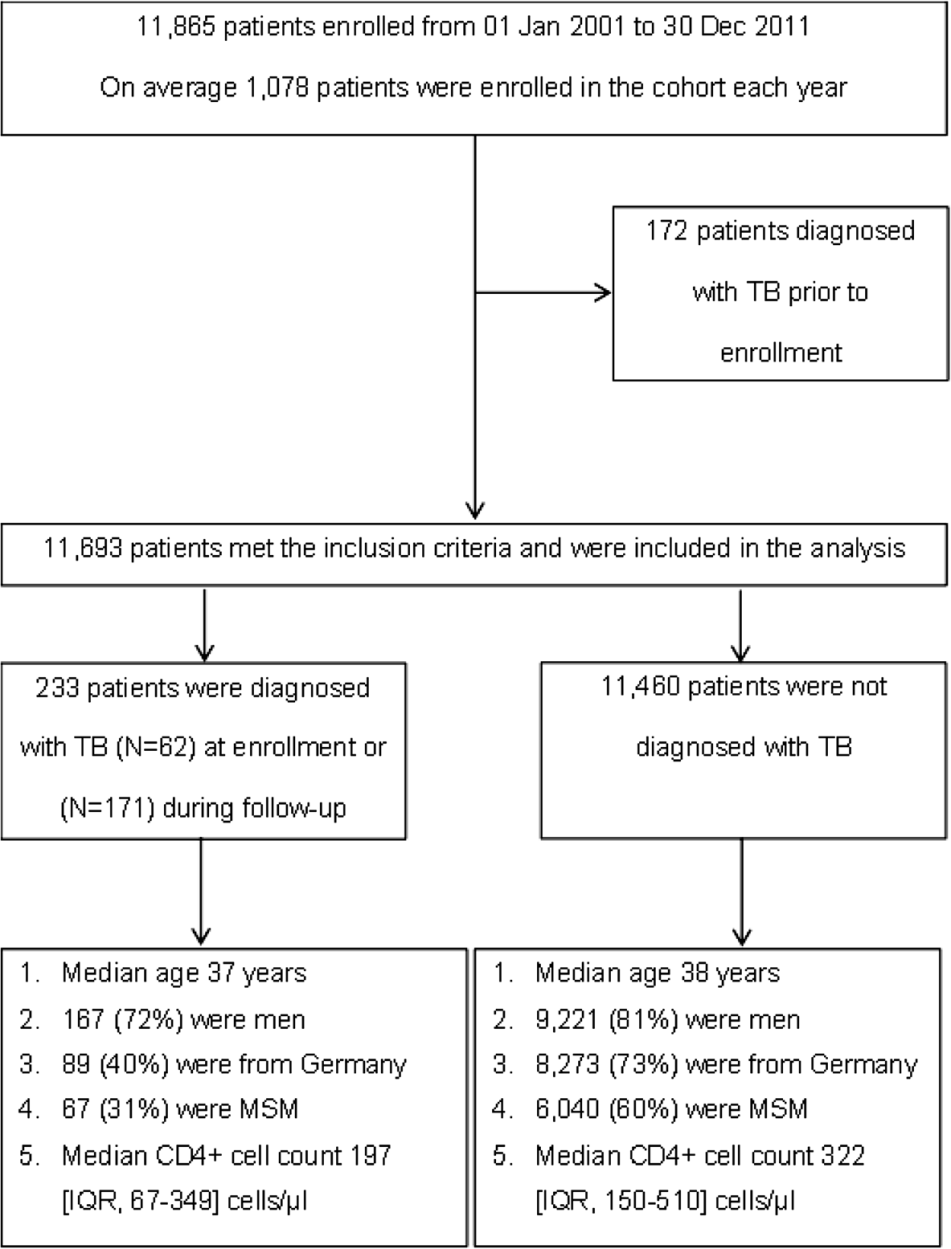
**Overview on the patients included in the study and their characteristics at enrollment in the ClinSurv HIV Cohort, Germany 2001–2011.** TB, tuberculosis; MSM, men who have sex with men; IQR, interquartile range.

From January 2001 through December 2011, a total of 11,865 patients were newly enrolled in the ClinSurv HIV cohort. Of them, 172 patients were known to have TB prior to enrolment and were therefore excluded from further analyses. Of a total of 11,693 eligible HIV-positive patients, 80% (N = 9,388) were men, 73% (N = 8,362) originated from Germany and 12% (N = 1,403) from Sub-Saharan Africa (Figure 1). About 80% of PLWHA received cART during the follow-up. Patients aged > 38 years and with low baseline CD4+ cell count and high viral load were more likely to receive cART (multivariable logistic regression; *P* < 0.05). No significant difference was found in the distribution of cART between patients from German and Sub-Saharan Africa (*P* = 0.79) (Table 1).

**Table 1:** Demographic and clinical characteristic of people living with HIV/AIDS by cART status in the ClinSurv HIV Cohort, Germany 2001–2011

**Characteristic**	**Non-cART**	cART*	**Total**
	**N = 2,467 (21.1%)**	**N = 9,226 (78.9%)**	**N = 11,693 (100%)**
TB patients	103 (4.2%)	130 (1.4%)	233 (2%)
Gender			
Male	1.994 (80.8%)	7,394 (80.2%)	9,388 (80.3%)
Female	473 (19.2%)	1,831 (19.9%)	2,304 (19.7%)
Age at enrollment, years			
Median [IQR]	35 [29–42]	39 [32–46]	38 [31–45]
≤38	1,468 (59.6%)	4,338 (47.1%)	5,806 (49.7%)
>38^†^	994 (40.4%)	4,873 (52.9%)	5,867 (50.3%)
Geographical origin			
Germany	1,731 (72.1%)	6,631 (73.0%)	8,362 (72.8%)
Sub-Saharan Africa	255 (10.6%)	1,066 (11.7%)	1,321 (11.5%)
HIV-transmission risk group			
MSM	1,339 (60.3%)	4,768 (57.8%)	6,107 (58.4%)
Other	881 (39.7%)	3,477 (42.8%)	4,358 (41.6%)
CD4+ cell count at enrollment in the cohort, cells/μl			
Median [IQR]	492 [349–663]	273 [118–453]	320 [146–508]
>200	2,054 (89.1%)	5,467 (61.9%)	7,521 (67.6%)
≤200^†^	251 (10.9%)	3,362 (38.1%)	3,613 (32.5%)
Viral load at enrollment in the cohort, log_10_ copies/ml			
Median [IQR]	4.2 [3.4 – 4.8]	4.5 [3.1 – 5.2]	4.4 [3.3 – 5.7]
<5	1,769 (72.9%)	5,845 (68.1%)	7,614 (70.5%)
≥5^†^	446 (20.1%)	2,737 (31.9%)	3,183 (29.5%)
Calendar year of enrollment			
2001	147 (5.9%)	765 (8.2%)	912 (7.7%)
2002	166 (6.7%)	832 (9.0%)	998 (8.4%)
2003	178 (7.2%)	839 (10.1%)	1,017 (8.7%)
2004	205 (8.3%)	928 (10.1%)	1,133 (9.7%)
2005	213 (8.6%)	908 (9.8%)	1,121(9.5%)
2006	225 (9.1%)	842 (9.1%)	1,067 (9.1%)
2007	251(10.2%)	893 (10.6%)	1,144 (9.8%)
2008	217 (8.8%)	883 (9.5%)	1,100 (9.4%)
2009	249 (10.1%)	835 (9.1%)	1,084 (9.3%)
2010	278 (11.3%)	811 (8.8%)	1,089 (9.3%)
2011	338 (13.7%)	690 (7.5%)	1,028 (8.8%)
Length of follow-up (person-years)			
Median [IQR]	1.2 [0.3-2.8]	3.1 [1.3-5.9]	2.5 [1.1-5.3]

cART, combination antiretroviral therapy; TB, tuberculosis; IQR; interquartile range; MSM, men who have sex with men; PWID, person who inject drugs; HPC, high HIV-prevalence countries.

*cART initiated during follow-up at any time prior to TB diagnosis.

^†^Significantly associated with receiving cART (multivariable logistic regression model including all presented characteristics as independent variables; *P* < 0.05).

Of 11,693 patients, 233 were diagnosed with TB (N = 62) at enrollment and (N = 171) during follow-up respectively. The majority of TB/HIV co-infected patients were male (167 cases (72%)). A large proportion of the female patients originated from Sub-Saharan Africa (41 cases (64% of 64 female patients)), while Germany was the main origin among male patients (81 cases (51% of 159 male patients)) (Table 2). At the time of enrollment, the median CD4+ cell count was significantly lower among patients who developed TB compared with those who did not (197 cells/μl [IQR; 67–349], 322 cells/μl [IQR; 150–510] respectively, *P* < 0.001). Only 130 (56%) patients of the 233 TB/HIV patients received cART at any point before TB diagnosis. A total of 25 TB/HIV co-infected patients had died during the investigation period (Table 2). Additional clinical characteristics were presented in Table 2.

**Table 2:** Demographic and clinical characteristic of people living with HIV/AIDS diagnosed with TB at enrollment and during follow-up in the ClinSurv HIV Cohort, Germany 2001–2011

**Characteristics**		**Female**	**Male**	**Total**
		**N = 66 (28.3%)**	**N = 167 (71.7%)**	**N = 233 (100%)**
Median age at enrollment (N = 232/1 missing data)	Median [IQR]	31 [26–45]	40 [32–47]	37 [30–45]
Median age at the time of TB diagnosis, years (N = 251/1 missing data)	Median [IQR]	32 [28–39]	40 [32–48]	37 [31–45]
Region of origin (N = 223/10 missing data)	N. (%)			
Germany		8 (12.5)	81 (50.9)	89 (39.9)
Sub-Saharan Africa		41 (64.1)	41 (25.8)	82 (36.8)
Other countries		15 (23.4)	37 (23.3)	52 (23.3)
HIV-transmission risk group (N = 214/19 missing data)	N. (%)			
MSM		---	67 (43.8)	67 (31.3)
PWID		3 (4.9)	17 (11.1)	20 (9.4)
HPC		54 (88.5)	43 (28.1)	97 (45.3)
Others^†^		4 (6.6)	26 (16.9)	30 (14.0)
Median CD4+ blood cell count at enrollment (N = 221/12 missing data)	Median [IQR]	197 [67–349]	204 [67–375]	189 [66–348]
Median CD4+ blood cell count at the time of TB diagnosis (N = 194/39 missing data)	Median [IQR]	211 [65–339]	198 [47–339]	216 [69–350]
Median viral load at enrollment (log_10_copies/ml) (N = 210/23 missing data)	Median [IQR]	4.8 [3.6-5.4]	4.7 [3.4-5.5]	4.9 [3.6-5.3]
Median viral load at the time of TB diagnosis (N = 145/88 missing data)	Median [IQR]	5.0 [4.3-5.6]	5.2 [4.5-5.7]	5.9 [3.9-5.5]
Hepatitis B* (N = 233)	Median [IQR]	25 (37.9)	39 (23.4)	64 (27.5)
Hepatitis C* (N = 233)	Median [IQR]	5 (7.6)	18 (10.8)	23 (9.9)
Diabetes mellitus* (N = 233)	Median [IQR]	2 (3.0)	5 (3.0)	7 (3.0)
cART^‡^ (N = 233)	Median [IQR]	31 (50.0)	99 (59.3)	130 (55.8)
Died (N = 233)	N. (%)	4 (10.6)	18 (10.8)	25 (10.7)

TB, tuberculosis; MSM, men who have sex with men; PWID, person who inject drugs; HPC, high HIV-prevalence countries; cART, combination antiretroviral therapy; IQR, interquartile range.

*At any time during follow-up.

^†^Others included heterosexual, blood transfusion and mother to child transmission.

^‡^cART initiated during follow-up at any time prior to TB diagnosis.

### TB incidence density rate during follow-up

The median duration of follow-up was 40 months [IQR, 14–76]. The overall TB IDR was 0.37 cases per 100 person-years [95% CI, 0.32-0.43] for a total observation of 45,698 person-years (Table 3).

**Table 3:** TB incidence density rate stratified by baseline demographic and clinical characteristics in the ClinSurv HIV Cohort, Germany 2001–2011

**Characteristics**	**No. of patients**	**Person-years**	**No. with TB**	**TB IDR [95% CI]**	***P *** value*
Total patients	11,631	45,698	170	0.37 [0.32-0.43]	
Gender					0.04
Female	2,283	9,153	45	0.49 [0.37-0.66]	
Male	9,347	36,536	125	0.34 [0.29-0.41]	
Median age, years					0.41 (NS)
≤38	5,773	23,250	91	0.39 [0.32-0.48]	
>38	5,838	22,387	78	0.35 [0.28-0.43]	
Region of origin					<0.001
Germany	8,344	33,449	71	0.21 [0.17-0.27]	
Sub-Sahara Africa	1,298	4,871	59	1.20 [0.94-1.56]	
Other countries	1,789	6,710	34	0.52 [0.38-0.71]	
HIV-transmission risk group category					<0.001
MSM	6,093	24,189	53	0.22 [0.17-0.28]	
HPC	1,528	5,910	69	1.17 [0.92-1.48]	
PWID	943	3,534	14	0.42 [0.23-0.67]	
Others^†^	1,846	7,768	22	0.28 [0.18-0.43]	
CD4+ blood cell count (cells/μl)					<0.001
>350	5,037	19,702	44	0.22 [0.17-0.30]	
200–350	2,459	9,893	37	0.36 [0.26-0.50]	
<200	3,580	14,111	82	0.58 [0.47-0.72]	
Viral load (log_10_ copies/ml)					<0.001
<5	7,583	30,137	92	0.31 [0.25-0.37]	
≥5	3,162	12,490	65	0.52 [0.41-0.66]	
Antiretroviral therapy					<0.001
Never started cART	2,424	4,801	59	1.23 [0.95-1.59]	
cART during follow-up	9,207	40,897	111	0.27 [0.23-0.33]	
Follow-up					<0.001
First year	2,421	10,174	109	1.07 [0.89-1.29]	
Second year	1,615	8,248	16	0.19 [0.12-0.32]	

TB, tuberculosis; IDR, incidence density rate (cases per 100 person-years); CI, confidence interval; NS, non-significant; MSM, men who have sex with men; HPC, high HIV-prevalence countries; PWID, person who inject drugs; cART, combination antiretroviral therapy.

*Obtained by χ^2^ test for difference in TB IDR.

^†^Others included heterosexual, blood transfusion and mother to child transmission.

The TB IDR was significantly higher among women compared with men (0.49 vs. 0.34 per 100PY, respectively; *P* = 0.04) and among patients originating from Sub-Saharan Africa compared with those from Germany (1.20 vs. 0.21 per 100PY, respectively; *P* < 0.001). However, controlling for region of origin revealed no significant difference in TB IDRs between women and men (Mantel-Haenszel test; *P* = 0.1).

The highest TB IDR was found among patients who never started cART compared with those on cART (1.3 vs. 0.3 cases per 100PY respectively; *P* < 0.001). Furthermore, significantly higher TB IDRs were found among patients with the following clinical characteristics: baseline viral load ≥5 log_10_ copies/ml and baseline CD4+ cell count <200 cells/μl. A significantly lower TB IDR was found among men who have sex with men (MSM) compared to other HIV-transmission risk groups (*P* < 0.001).

The TB IDR was still significantly higher among patients with the aforementioned characteristics after controlling for age groups (Mantel-Haenszel test).

### Trend in TB incidence

**Figure 2:**
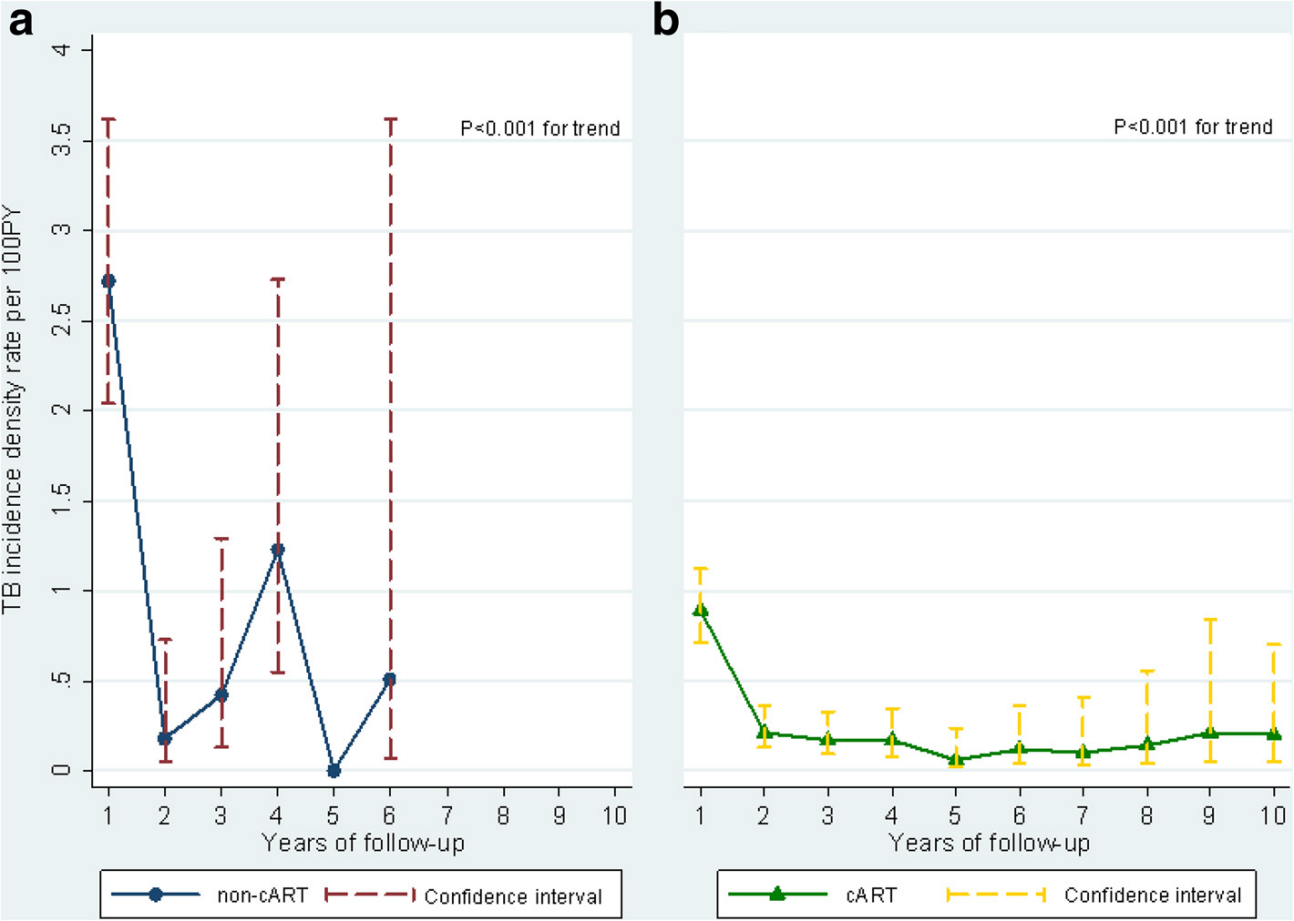
**The trend of tuberculosis incidence density rate during follow-up, among (a) patients who never started cART (N = 59) and (b) patients on cART (N = 111) in the ClinSurv HIV Cohort, Germany 2001–2011.** A low number of patients who never started cART remained under observation beyond 4 years of follow-up, where no TB cases reported in the 5th, 7th, 8th, 9th and 10th years of follow-up. *P* < 0.001 for trend for TB incidence in patients who never started cART and patients on cART.

The TB IDR was highest in the first year of follow-up at 1.07 per 100PY and decreased markedly in the second year to 0.19 per 100PY (*P* < 0.001) (Table 3). A significant reduction in the IDR was indicated during the follow-up for both patients on cART and those who never started cART (*P* < 0.001 for trend) (Figure 2). TB IDRs among patients on cART decreased further after the second year, while TB IDRs among those who never started cART tended to fluctuate and remained substantial (Figure 2).

### Kaplan-Meier survival probability estimates

**Figure 3:**
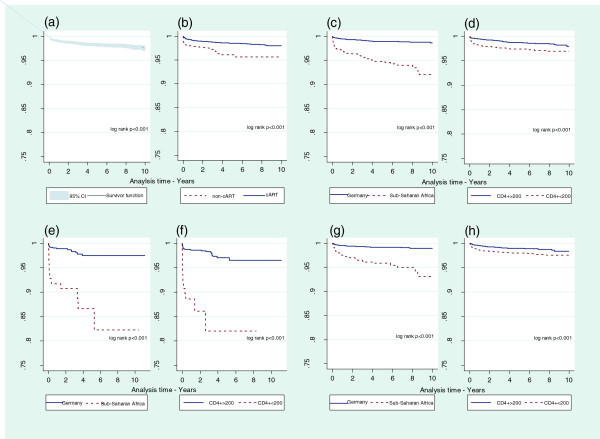
**Kaplan-Meier plots of tuberculosis (TB)-free survival proportion in the ClinSurv HIV Cohort, Germany 2001–2011. (a)** among all patients; **(b)** among all patients stratified by combination antiretroviral therapy (cART)-status; **(c)** among all patients stratified by region of region; **(d)** among all patients stratified by CD4+ cell count; **(e)** among patients who never started cART stratified by region of origin; **(f)** among patients who never started cART stratified by CD4+ cell count; **(g)** among patients on cART stratified by region of origin; **(h)** among patients on cART stratified by CD4+ cell count. Observation period for patients who never started cART began at enrollment, while for patients on cART began at the time of cART initiation.

The TB-free survival probability for the total cohort over the 10-year follow-up was 97% [95% CI 96%-98%] (Figure 3a). The 10-year TB-free survival proportion was significantly higher among patients on cART compared with those who never started cART (98% and 95%, respectively; *P* < 0.001) (Figure 3b). Among patients on cART, a significant lower TB-free survival proportion was found in patients originating from Sub-Saharan Africa compared with patients from Germany (93% and 99%, respectively; *P* < 0.001) (Figure 3c) and in patients with CD4+ cell count ≤200 cells/μl compared with patients with CD4+ cell count >200 cells/μl (98% and 99%, respectively; *P* < 0.001) (Figure 3d). The lowest TB-free survival proportion was among patients who never started cART, had a CD4+ cell count ≤200 cells/μl and originating from Sub-Saharan Africa (60%; *P* < 0.001) (data not shown).

### Risk factors for tuberculosis

In the multivariable analyses including gender, region of origin, HIV-transmission groups, CD4+ cell count and viral load as explanatory variables; risk factors of TB in patients who never started cART were: originating from Sub-Saharan Africa (Hazard ratio (HR) 4.05; 95% CI 1.87-8.78; *P* < 0.001), CD4+ cell coun t ≤ 200 cells/μl (HR 8.22; 95% CI 4.36-15.51; *P* < 0.001) and viral load ≥5 log_10_ copies/ml (HR 2.51; 95% CI 1.33-4.75; *P* = 0.005) (Table 4).

**Table 4:** Cox proportional hazards analysis of factors associated with TB among people living with HIV/AIDS in the ClinSurv HIV Cohort, Germany 2001–2011

**Predictor variable**	Patients never started cART*		**Patients on cART**^**†**^	
	**HR [95% CI]**	***P *** **value**	**HR [95% CI]**	***P *** **value**
Gender				
Female	1		1	
Male	0.73 [0.36-1.47]	0.38 (NS)	1.49 [0.84- 2.62]	0.17 (NS)
Median age, years				
≤38	1		1	
>38	0.86 [0.47-1.58]	0.62 (NS)	1.42 0.89 2.27	0.14 (NS)
Region of origin				
Germany	1		1	
Sub-Saharan Africa	4.05 [1.87-8.78]	<0.001	5.15 [2.76-9.60]	<0.001
Other countries	1.75 [0.81-38.1]	0.16	2.22 [1.18-4.20]	0.014
HIV-transmission risk group				
Others^†^	1		1	
MSM	0.68 [0.31-1.51]	0.34	0.60 [0.32-1.10]	0.10 (NS)
CD4+ cell count (cells/μl)				
≥200	1		1	
<200	8.22 [4.36-15.51]	<0.001	1.90 [1.14-3.15]	0.013
Viral load (log_10_ copies/ml)				
< 5	1		1	
≥ 5	2.51 [1.33-4.75]	0.005	1.77 [1.11-2.82]	0.016

HR, hazard ratio; CI, confidence interval; NS, non-significant; MSM, men who have sex with men.

*50 TB cases among 1,919 subjects. ^†^78 TB cases among 6,357 subjects.

^†^Others included heterosexual, blood transfusion and mother to child transmission.

Patients on cART shared the same risk factors of TB with the patients who never started cART, namely: originating from Sub-Saharan Africa (HR 5.15; 95% CI 2.76-9.60; *P* < 0.001), CD4+ cell count ≤ 200 cells/μl (HR, 1.90; 95% CI 1.14-3.15; *P* = 0.013) and viral load ≥ 5 log_10_ copies/ml (HR 1.77; 95% CI 1.11-2.82; *P* = 0.016). Additionally, other geographic origins were significantly associated with TB risk in patients on cART (Table 4).

Although MSM was negatively associated with the diagnosis of TB in univariable analyses (data not shown), it was not associated with risk of TB in the multivariate analyses. Gender and age were also not independently associated with TB risk in PLWHA in both models.

## Discussion

This study investigated the long-term TB incidence and risk factors among PLWHA in Germany. A specific feature of the study is that it is not restricted to patients receiving cART, but determines TB IDR and TB risk factors also for patients who never started cART. The ClinSurv HIV Cohort used in this study is characterized by a long duration of follow-up and broad enrollment criteria of PLWHA irrespective of their age, disease stage or ART-status. Therefore, this instrument allowed us to calculate long-term TB incidence rate and to determine associated risk factors with high precision and to update existing estimates [20]. This enhances the generalizability of the study results to other countries with low TB and HIV incidence.

Of 11,693 patients enrolled in the ClinSurv HIV Cohort, 233 were diagnosed with TB. Nearly 27% (N = 62) of TB cases were diagnosed at enrollment representing prevalent TB at time of clinic entry rather than true incident TB; a diagnosis of these TB episodes may have promoted HIV testing. These TB cases could potentially have been averted if individuals have been offered early HIV testing and enrolled early in a treatment center.

The TB IDR among HIV-positive patients in this study was 0.37 per 100PY; this result is consistent with findings from other studies conducted in high-income countries. A study from France showed that TB IDR was 0.4 per 100PY [6] and a study from the United Kingdom found that TB IDR was 0.3 per 100PY [4].

Previous studies attributed the great reduction of TB incidence, in the short term, to cART initiation [21-23]. In our study, we demonstrate a significant decrease in TB incidence density rate also among patients who never started cART during the second year. Therefore, the short-term impact of cART on TB incidence should be interpreted with caution. A higher TB rate in the first year could be due to late HIV diagnosis or closer TB monitoring in HIV-infected patients. However, the present data show that the TB incidence remained low during 10 years among patients on cART. This result is in agreement with findings from a recent large study (the CASCADE cohorts) [24]. Nevertheless, among patients on cART the TB rate did not further decrease in the long term. This is consistent with the immunological fact that cART has limitations in normalizing immune cell phenotype and function and fails to reduce the TB incidence to a level similar to that in an HIV-negative population [12,25]. It is not clear why a significant reduction in TB incidence rate among patients who never started cART occurred in the second year. One explanation could partly be a result of survival bias, as the majority of deaths in the cohort (47%) occurred in the first year of observation. The low number of patients who never started cART as well as low TB frequency among them beyond 4 years of follow-up prevented comparison of the TB trend in relation to cART beyond this time point.

The data from our study confirm that originating from Sub-Saharan Africa was independently associated with increased TB risk. Similar findings were reported in another study done in France, where the relative risk of TB was 2.16 among PLWHA originating from Sub-Saharan Africa compared with patients born in France [6]. These findings can be explained in light of the high latent TB infection (LTBI) prevalence and TB incidence in their country of origin [2]. Furthermore, a wide range of socio-economic, cultural and legal factors may also play a role in increased vulnerability to TB among immigrants in term of high-risk behavior and barriers to health care services [26], even though access to TB and HIV care is supposed to be free in Germany. The presumably high LTBI prevalence among PLWHA originating from Sub-Saharan Africa might expose them to a particular risk of IRIS triggered by host immune responses restored after the initiation of cART [27,28].

Consistent with other findings from studies done in developing and industrialized countries [6,21-24,29,30], lower CD4+ cell count and higher viral load level at enrollment were independently associated with higher risk for TB.

Both patients on cART and those who never started cART share the same risk factors for TB. This evidence highlights the need for early screening of LTBI and offering isoniazid preventive therapy (IPT); particularly to patients originating from Sub-Sahara Africa and those with poor immune status. In the ClinSurv HIV Cohort only 0.1% of 11,693 PLWHA without TB were reported to receive isoniazid (data not shown). This might be partly related to incomplete recording of this information but could also indicate low level of IPT implementation in Germany. A similar finding was shown by a French study, which showed that IPT was rarely implemented in France in PLWHA [6].

IPT can dramatically reduce the risk of TB among PLWHA even among those receiving ART and living in areas with low TB rates [31-33]. The new WHO guideline strongly recommends IPT to HIV patients without active TB irrespective of immune status and whether or not a person is on ART [32]. The British HIV Association Guideline identified HIV patients with increased risk for TB as being from sub-Saharan Africa, with CD4+ cell count <350 cells/μl, or duration of cART <6 months and recommended that these patient groups should be offered screening for LTBI and given TB chemoprophylaxis if the test result is positive [34]. Our findings support these recommendations and we therefore recommend a similar approach to be implemented in Germany.

This study has several limitations. A geographical bias related to distribution of collaborating treatment centres of the ClinSurv HIV Cohort can be noticed; cities with a relatively high notification rate of TB and HIV in Germany like Stuttgart, Frankfurt and Nuremberg [9] are left without local collaborating centres [17]. The collaborating centres are mainly specialized to treat patients with advanced disease stage, thus patients with HIV C-classification, including those with TB, might be overrepresented in the cohort. Bätzing-Feigenbaum et al. [17] also found that patients originating from high HIV-prevalence countries were slightly underrepresented in the ClinSurv HIV Cohort compared to national HIV surveillance data, which could be an expression of greater difficulty in accessing specialized treatment centres. This implies that this study underestimates the actual TB/HIV burden in Germany. It is unknown whether TB diagnosis was routinely implemented to all PLWHA (active case finding) or whether it was just a part of on-going health care (passive case finding); therefore some TB cases may not have been detected.

Discussion is also necessary on whether the TB case definition in the ClinSurv HIV Cohort (culture-positive) is strictly implemented, which might underestimate the TB burden. Some TB cases could have also been clinically diagnosed and recorded in the ClinSurv HIV Cohort (yet was not provable case-by-case), because these TB cases are also mandatorily notified within the German TB surveillance. Due to lack of data, we were unable to include some factors known to be associated with TB in our analysis such as smoking [35], homelessness [36], alcohol abuse [37], incarceration [38] and other socio-economic factors.

## Conclusion

In conclusion, the German ClinSurv HIV Cohort represents a highly valuable data source to study the occurrence of TB/HIV in Germany. A reasonable amendment to the instrument would be the systematic collection of data on LTBI and TB diagnostics done and the completion of data on IPT.

Particularly, patients originating from Sub-Saharan African countries with high incidence of TB, and those with low CD4+ cell count and high viral load were at increased risk of TB even after cART initiation. This suggests that actions beyond timely cART initiation should be considered; including early screening for LTBI and offering IPT in line with available recommendations.

## Abbreviations

HIV: Human immunodeficiency virus; AIDS: Acquired immunodeficiency syndrome; PLWHA: People living with HIV/AIDS; cART: Combination antiretroviral therapy; TB: Tuberculosis; IRIS: Immune reconstitution inflammatory syndrome; NRTI: Nucleoside reverse transcriptase inhibitor; NNRTI: Non-nucleoside reverse transcriptase inhibitor; CDC: Centres for disease control and prevention; IQR: Interquartile rang; IDR: Incidence density rate; PY: Person-year; CI: Confidence interval; MSM: Men who have sex with men; HPC: High HIV-prevalence countries; PWID: Person who inject drugs; HR: Hazard ratio; LTBI: latent TB infection; IPT: Isoniazid preventive therapy

## Competing interests

The authors declare that they have no competing interests.

## Authors’ contributions

Concept and design (LF, BK, WH), statistical analyses (BK), interpretation of the data (BK, WH, CK, BGB, OH, LF), drafting the manuscript (BK) and critical revision of the manuscript for important intellectual content (WH, CK, BGB, OH, LF). All authors read and approve the final manuscript.

## Authors’ information

The German ClinSurv HIV Study Group: Berlin: Robert Koch Institute: A. Kühne (Cohort manager); Vivantes Auguste-Viktoria-Hospital: K. Arastéh; Charité University Medicine: F. Bergmann, M. Warncke; Bochum: St.-Josef-Hospital/Ruhr University: N. Brockmeyer, N. Mühlbächer; Bonn: University Hospital: J. Rockstroh, J. Wasmuth, S. Hass; Düsseldorf: University Hospital Düsseldorf: B. Jensen, L. Rollmann; Essen: University Hospital Essen: S. Esser, P. Schenk-Westkamp; Hamburg: ifi-Institute for Interdisciplinary Medicine; A. Plettenberg, F. Kuhlendahl; ICH Studycenter: A. Adam, L. Weitner, K. Schewe, H. Goey, S. Fenske, T. Buhk, H.-J. Stellbrink, C. Hoffmann; University Hospital Eppendorf: J. van Lunzen, K. Wassmus; Hanover: Medical University Hanover: M. Stoll, S. Gerschmann, K. Hoeper; Kiel: University Schleswig-Holstein, Campus Kiel: H.A. Horst, S. Trautmann; Cologne: University Hospital Cologne: G. Fätkenheuer, D. Gillor, P. Schommers; Munich: University Hospital, Ludwig-Maximilian University: J. Bogner, B. Sonntag; Regensburg: University Hospital Regensburg: B. Salzberger; Rostock: University Hospital Rostock: C. Fritzsche

## Pre-publication history

The pre-publication history for this paper can be accessed here:

http://www.biomedcentral.com/1471-2334/14/148/prepub
